# Significant Regional Differences in Lung Cancer Incidence in Hungary: Epidemiological Study Between 2011 and 2016

**DOI:** 10.3389/pore.2021.1609916

**Published:** 2021-09-14

**Authors:** Gabriella Gálffy, Aladár Vastag, Krisztina Bogos, Zoltán Kiss, Gyula Ostoros, Veronika Müller, László Urbán, Nóra Bittner, Veronika Sárosi, Zoltán Polányi, Zsófia Nagy-Erdei, Andrea Daniel, Kata Knollmajer, Máté Várnai, Péter Szegner, Zoltán Vokó, Balázs Nagy, Krisztián Horváth, György Rokszin, Zsolt Abonyi-Tóth, Éva Pozsgai, Zsófia Barcza, Judit Moldvay, Lilla Tamási

**Affiliations:** ^1^ Department of Pulmonology, Pulmonology Hospital Törökbálint, Törökbálint, Hungary; ^2^ MSD Pharma Hungary Ltd, Budapest, Hungary; ^3^ Department of Pulmonology, National Korányi Institute of Pulmonology, Budapest, Hungary; ^4^ Department of Pulmonology, Semmelweis University, Budapest, Hungary; ^5^ Department of Pulmonology, Mátraháza Healthcare Center and University Teaching Hospital, Mátraháza, Hungary; ^6^ Pulmonology Clinic, University of Debrecen, Debrecen, Hungary; ^7^ Department of Pulmonology, Faculty of Medicine, University of Pécs, Pécs, Hungary; ^8^ Center for Health Technology Assessment, Semmelweis University, Budapest, Hungary; ^9^ Health Services Management Training Centre, Semmelweis University, Budapest, Hungary; ^10^ Department of Health Policy and Health Economics, Eötvös Loránd University, Budapest, Hungary; ^11^ RxTarget Ltd., Szolnok, Hungary; ^12^ Department of Biostatistics, University of Veterinary Medicine Budapest, Budapest, Hungary; ^13^ Department of Primary Health Care, University of Pécs Medical School, Pécs, Hungary; ^14^ Syntesia Medical Communications Ltd., Budapest, Hungary; ^15^ Department of Tumor Biology, National Korányi Institute of Pulmonology, Semmelweis University, Budapest, Hungary; ^16^ MTA-SE NAP, Brain Metastasis Research Group, Hungarian Academy of Sciences, 2nd Department of Pathology, Semmelweis University, Budapest, Hungary

**Keywords:** epidemiology, Hungary, mortality, lung cancer, incidence, Hungarian regions

## Abstract

**Objective:** Hungary has one of the highest incidences and mortality rates of lung cancer (LC), therefore the objective of this study was to analyse and compare LC incidence and mortality rates between the main Hungarian regions.

**Methods:** This nationwide, retrospective study used data from the National Health Insurance Fund and included patients aged ≥20 years who were diagnosed with lung cancer (ICD-10 C34) between Jan 1, 2011 and Dec 31, 2016. Age-standardized incidence and mortality rates were calculated and compared for the main regions.

**Results:** The highest incidence rate in males was recorded in Northern Hungary (146.8/100,000 person-years [PY]), while the lowest rate was found in Western Transdanubia (94.7/100,000 PY in 2011). All rates showed a declining trend between 2011 and 2016, with the largest decrease in the Northern Great Plain (−20.0%; *p* = 0.008). LC incidence and mortality rates in women both showed a rising tendency in all regions of Hungary, reaching the highest in Central Hungary (59.86/100,000 PY in 2016). Lung cancer incidence and mortality rates in males correlated with the level of education and smoking prevalence (*p* = 0.006 and *p* = 0.01, respectively) in the regions. A correlation with GDP per capita and Health Development Index (HDI) index could also be observed in the Hungarian regions, although these associations were not statistically significant. No correlations could be detected between these parameters among females.

**Conclusion:** This analysis revealed considerable differences in the epidemiology of LC between the 7 main Hungarian regions. LC incidence and mortality rates significantly correlated with smoking and certain socioeconomic factors in men, but not in women. Further research is needed to explain the regional differences.

## Background

Lung cancer (LC) accounts for almost 1 in 5 cancer-related deaths and is therefore a leading cause of cancer worldwide, including Eastern Europe [1]. Based on WHO GLOBOCAN data, one of the highest global age-standardized incidence rates in both genders was found in Hungary (1). The report by Ferlay et al. estimated the incidence of LC to be 111.6/100,000 standardized person-years (PY) among men and 58.7/100,000 PY among women in Hungary in 2018, using the ESP 1976 for standardization (2). Mortality rates were similarly prominent in Hungary, with an age-standardized mortality rate as high as 96.4/100,000 PY in men and 37.7/100,000 PY in women in 2012. A downward trend over time could be observed in LC incidence among Hungarian men, but an increase in incidence among Hungarian women [2].

Despite the leading LC rates reported in previous publications, results from a recent retrospective study suggest that although Hungarian LC incidence and mortality rates are high, they are likely to be lower than previously described. The report showed a decrease in the age-adjusted incidence rate of LC in Hungary from 115.7 to 101.6/100,000 PY in men, and an increase from 48.3 to 50.3/100,000 SPYs in women between 2011 and 2016 (using ESP1976) [3]. The differences between previous reports could be attributed to the use of different data sources: while earlier studies used WHO GLOBOCAN annual reports which received mortality data from the Hungarian Central Statistical Office (CSO), this recent study gathered more accurate data on LC incidence and mortality among patients with LC from the comprehensive Hungarian National Health Insurance Fund (NHIF) database.

Risk factors for LC include environmental and lifestyle factors, with tobacco smoking being the most important [4,5]. LC rates are massively influenced by tobacco exposure[6,7], giving rise to 20-fold variations between LC rates by regions. Socioeconomic status has also been shown to be associated with LC incidence and mortality. Wong et al. found that countries with higher levels of Human Development Index (HDI), which comprises life expectancy at birth, access to knowledge and income per capita adjusted for purchasing-power parity, had higher LC incidence and mortality [8,9]. Considerable differences were found in LC incidence and mortality rates between different Surveillance, Epidemiology, and End Results (SEER) sites in the U.S., suggesting that regional differences may be influenced by environmental factors, among others [10,11].

The large differences in LC incidence and mortality rates between geographical regions, and the overall leading role of LC in cancer-related deaths worldwide including Hungary emphasize the importance of obtaining accurate and more detailed data regarding the epidemiology of LC. The implementation and testing of preventive public health strategies and new treatments can only be efficient with the availability of reliable data.

To date, no study has been conducted to evaluate epidemiological differences in LC between the seven main regions of Hungary (Central Hungary, Northern Great Plain, Southern Great Plain, Northern Hungary, Central Transdanubia, Southern Transdanubia, and Western Transdanubia). Therefore, the objective of this study was to analyse LC incidence and mortality rates in the main regions of Hungary, to observe and compare changes, and to identify possible trends between 2011 and 2016. We also aimed to investigate whether there was a correlation between the incidence and mortality of LC and Gross Domestic Product (GDP) per capita, levels of income and education, Health Development Index (HDI) and smoking prevalence in the seven main Hungarian regions.

## Materials and Methods

### Study Design

This nationwide retrospective, longitudinal study was conducted using the database of the National Health Insurance Fund of Hungary (NHIF). The NHIF database is a nationwide insurance system covering close to 100% of the Hungarian population, which collects patient ID and ICD-10 code records about all in- and out-patient visits and all prescription of drugs which are reimbursed in Hungary. The study was approved by the National Ethical Board for Health Research (10338-5/2019/EKU).

We enrolled patients who were newly diagnosed with lung cancer (ICD-10:C34) between Jan 1, 2011 and Dec 31, 2016 and who were above 20 years of age at the time of diagnosis.

In order to identify newly diagnosed LC patients from 2011, we set a reference screening period for 2009–2010. Potential miscoding of lung cancer was prevented by only including patients with a minimum of two records of the C34 ICD-10 code within an interval of over 30 but less than 365 days following the initial coding. Patients with only one recorded C34 code who died within 60 days after coding were also included. Patients were excluded if they had ICD-10 codes related to other cancers or if they were treated with oncological therapies other than the lung cancer-specific treatment protocol 6 months prior to or 12 months following the first recorded lung cancer code. Since the immediate cause of death was not available from the NHIF database, all-cause mortality data were accessed. During data collection, data were anonymised, and only non-identifiable data were used in the investigation.

The annual numbers of newly diagnosed lung cancer patients are presented as crude numbers (n). Annual incidence rates are expressed as standardized rates per 100,000 person-years. Data regarding population sizes for incidence rate calculations by age and sex were obtained from the public annual reports of the Hungarian Central Statistical Office. The dates of LC patient’s deaths and the number of deceased patients per year were acquired from the National Health Insurance Fund database. All-cause mortality was expressed as crude numbers and all-cause mortality rates per standardized rates of 100,000 person-years. During standardization, all crude incidence and mortality data were adjusted for age using the European Standard Population from 2013, however standardization was performed from the 1976 European Standard Population as well, to enable comparisons with European countries from earlier publications. Where crude numbers of any parameter were below 10, we indicated “<10” since the Hungarian NHIF data protection laws prohibit the presentation of case numbers below 10. In such cases, calculations were based on the exact crude numbers. All incidence and mortality rates were calculated for the seven main regions of Hungary (Central Hungary, Northern Great Plain, Southern Great Plain, Northern Hungary, Central Transdanubia, Southern Transdanubia, and Western Transdanubia).

### Statistical Analysis

Population at risk was estimated from the mid-year population minus the number of previously diagnosed LC patients who were alive at 1 January of each year. We were using Poisson regression to estimate standardised LC incidence and mortality rates. Standardised rates/100,000 person-years using the European Standard Population from 2013 were used, the explanatory variables were the year, region and their interactions. As data corresponding to different years were not independent, bootstrap time series with a fixed block size of 2 with 1,000 repetitions was used for the regression models estimate the confidence intervals of the rate ratios. We plotted standardised mortality rates and standardised incidence rates for European countries alongside analogous data published by Ferlay et al. [2]. The aim was to demonstrate the position of Hungarian regions in the spectrum of incidence and mortality compared to European countries.

The correlation analysis of age-standardised incidence and mortality rates in 2013 with smoking prevalence (in 2003), Health Development Index (in 2003), income level (in 2003), education level (in 2003) and GDP per capita (in 2003) was performed, as well. We had aggregated values for the seven regions. Their rank-correlation was analysed using Kendall’s tau. All calculations were performed with R version 3.5.2 (2018-12-20) with package boot version 1.3–20.

## Results

### LC Incidence and Mortality Rates in Hungarian Regions

**FIGURE 1:**
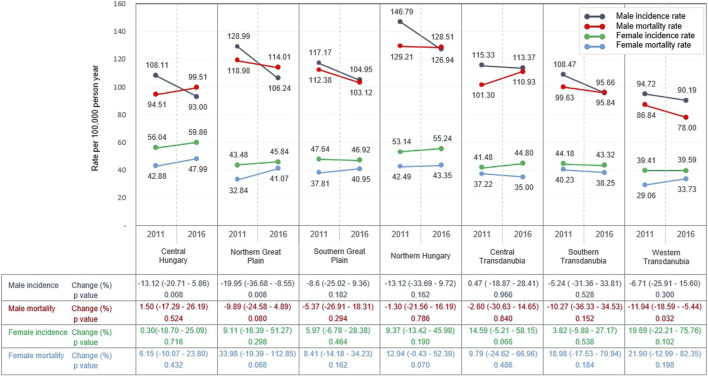
Standardised LC incidence and mortality rates per 100,000 person-years in 2011 and in 2016 in the main regions of Hungary (using ESP 2013 population) (rates in 2011 and 2016 represents the actual data, while changes of rates were calculated with Poisson regression using all rates recorded yearly between 2011 and 2016).

Crude incidence and mortality are presented in Table 1 by region and year. The highest standardised (for ESP 2013) incidence rates in men were recorded in Northern Hungary both in 2011 and 2016 (146.8/100,000 PY and 126.9/100,000 PY, respectively) (Figure 1 and Supplementary Tables S1,2). The lowest male incidence rates were found in Western Transdanubia both in 2011 and 2016 (94.7/100,000 PY and 90.2/100,000 PY, respectively). Male LC incidence showed a declining trend between 2011 and 2016, and the largest and significant decrease in incidence was detected in the Northern Great Plain (−20.0%; 95% CI: −36.7 to −8.6; *p* = 0.008). Similar to incidence rates, the highest male mortality rates were also found in the Northern Hungarian region (129.2/100,000 PY in 2011 and 128.5/100,000 PY in 2016), and the lowest mortality rates in Western Transdanubia (86.8/100,000 PY in 2011 and 78.00/100,000 PY in 2016). There was a declining trend during the study period in most regions, except for Central Hungary (1.5%; 95% CI: −17.3–26.2; *p* = 0.5), where male mortality rates increased slightly.

**TABLE 1:** Lung cancer patients in Hungary by regions between 2011 and 2016 (Newly diagnosed and number of lung cancer patients died).

	Number of patients					
Patients with new LC diagnosis	2011	2012	2013	2014	2015	2016
Total country (n)	7,158	6,924	6,856	6,949	6,981	6,996
Regions (n)						
Central Hungary	2,106	2,133	2,056	2,050	2,035	2,114
Northern Great Plain	1,050	1,066	1,066	1,015	1,028	986
Southern Great Plain	977	953	984	1,003	989	927
Northern Hungary	1,044	884	909	954	852	965
Central Transdanubia	728	721	709	739	786	770
Southern Transdanubia	656	624	605	624	690	620
Western Transdanubia	597	543	527	564	601	614

Number of patients died	2011	2012	2013	2014	2015	2016
Total country (n)	6,045	6,208	6,154	6,283	6,273	6,465
Regions (n)						
Central Hungary	1,711	1,860	1,817	1,825	1,869	1,964
Northern Great Plain	878	922	951	985	948	972
Southern Great Plain	857	896	862	891	915	861
Northern Hungary	868	791	806	884	805	871
Central Transdanubia	649	639	638	672	629	694
Southern Transdanubia	590	591	566	534	579	586
Western Transdanubia	492	509	514	492	528	517

LC incidence and mortality rates in women showed different tendencies, since both increased in all regions of Hungary. The highest female LC incidence was registered in Central Hungary (56.0/100,000 PY in 2011 and 60.0/100,000 PY in 2016), and the lowest incidence in the Western Transdanubian region (39.4/100,000 PY in 2011 and 39.6/100,000 PY in 2016). The greatest, but not significant change in incidence was observed in Western Transdanubia (+19.7%; 95% CI: −22.2–75.8; *p* = 0.1). Like incidence rates, female mortality rates increased during the study period, with the highest mortality rates observed in Central Hungary and the lowest in Western Transdanubia. The greatest increase in LC mortality among women in the study period was observed in the Northern Great Plain (+34.0; 95% CI: −19.4–112.9; *p* = 0.07), however, this increase was not statistically significant, either (Figure 1 and Supplementary Tables S1,2).

### Hungarian Regional LC Incidence and Mortality Rates Compared With Ferlay’s 2012 Eu Ranking of LC Incidence and Mortality Rates

**FIGURE 2:**
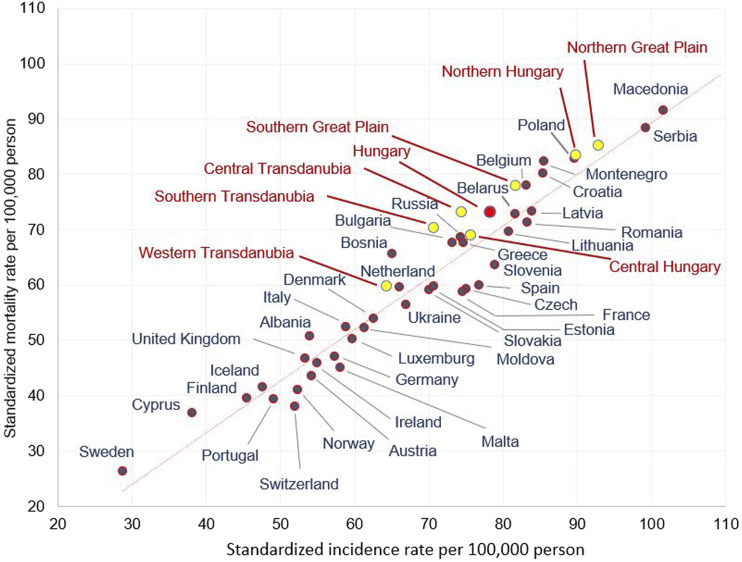
Age-standardised incidence and mortality rates (per 100.000 person years) in males in European countries and in the Hungarian regions in 2012 (using European standard population 1976).

In Figure 2, the axes represent standardised incidence rates and standardized mortality rates per 100,000 population in males, respectively.

In the Western Transdanubian region, where the male incidence and mortality rates were the lowest in Hungary, the figures were comparable to some Western European countries (e.g., the Netherlands). On the other hand, in Northern Hungary and the Northern Great Plain, incidence and mortality rates in males stood out and were similar to certain Eastern- and South-Eastern European countries (e.g., Poland, Serbia) (Figure 2). A similar variability of mortality and incidence rates of different Hungarian regions could also be observed in females, although to a lesser degree. The lowest mortality and incidence rates registered in Western Transdanubia were similar to Albania and Germany, while the highest rates found in Central Hungary were comparable to Iceland (Supplementary Figure S1)

### LC Risk Compared to the Central Hungarian (Reference) Region

**FIGURE 3:**
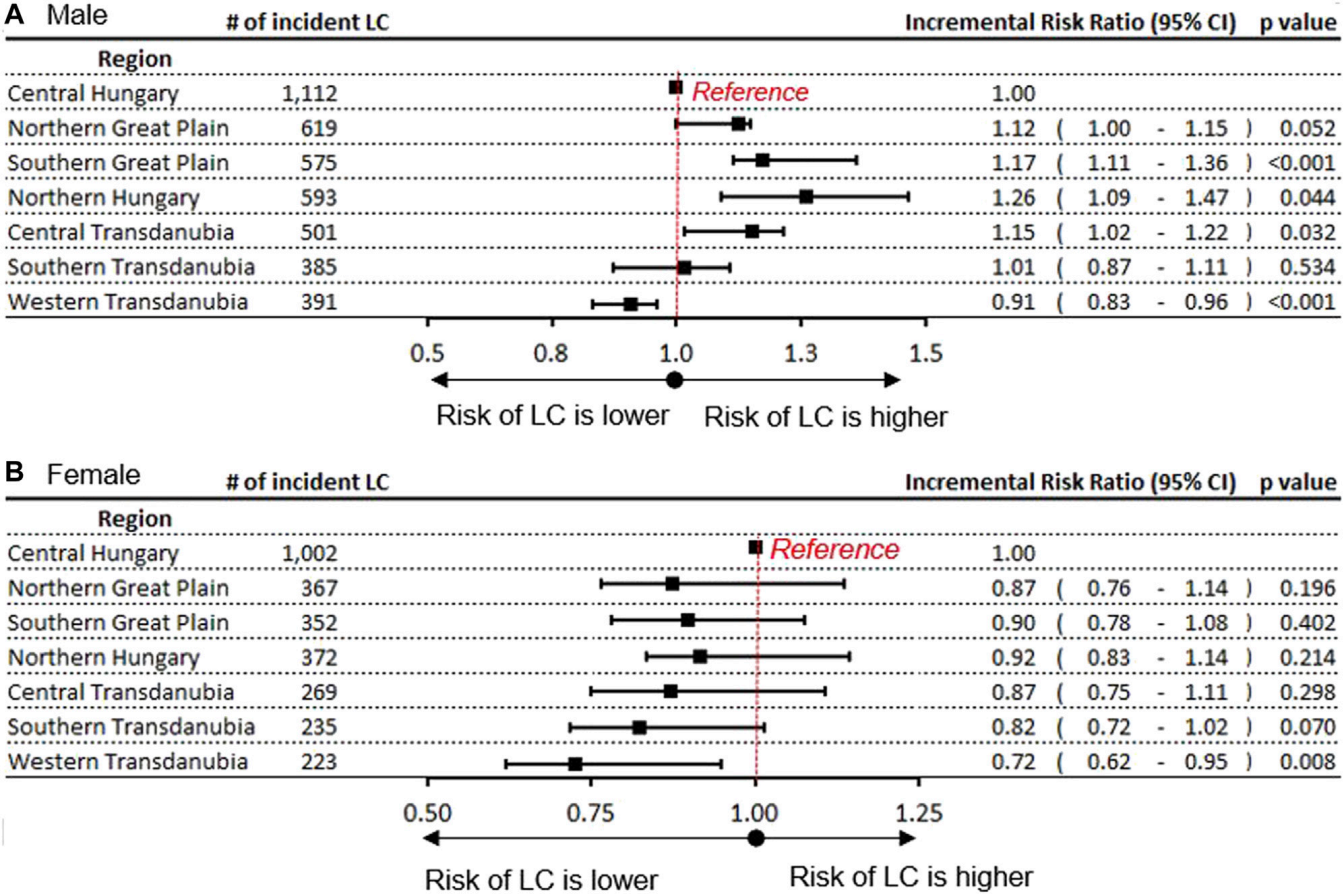
Incidence rate ratio of LC in Hungary’s regions with the Central Hungarian region as reference in **(A)** males **(B)** females.

The relative rate of LC was evaluated in the study year 2016 using the Central Hungarian region as a reference (Figure 3A). We found a significantly higher rate of LC among men in Northern Hungary (rate ratio 1.26; 95% CI:1.09–1.47; *p* = 0.04). Significantly higher LC risks could also be detected in the Southern Great Plain (1.17) and in Central Transdanubia (1.15). The incidence rate in males was lower in Western Transdanubia (0.85; 95% CI: 0.72–0.88; *p* < 0.0001) compared to Central Hungary in 2016. This tendency of risk distribution was apparent in 2011 as well, although the differences were larger (Supplementary Figure S2). The greatest differences between the highest and lowest LC risks were found in Northern Hungary and Western Transdanubia in both studied years, with a rate ratio of 1.42 in 2011 and 1.35 in 2016 (Figure 3A and Supplementary Figure S2).

In contrast, the risks of LC in females were the highest in the reference, Central Hungarian region both in 2011 and 2016. Similar to males, the lowest risks of LC in females were found in Western Transdanubia in both 2011 0.61; 95% CI: 0.45–0.80; *p* = 0.02) and 2016 (0.72; 95% CI: 0.62–0.95; *p* = 0.008) (Figure 3B and Supplementary Figure S3).

LC mortality in Hungary’s regions is provided as additional data in Supplementary Figure S3. Regarding regional and gender differences, mortality showed a similar distribution to incidence risks.

### The Relationship Between LC Incidence and Mortality Rates and Smoking Prevalence

**FIGURE 4:**
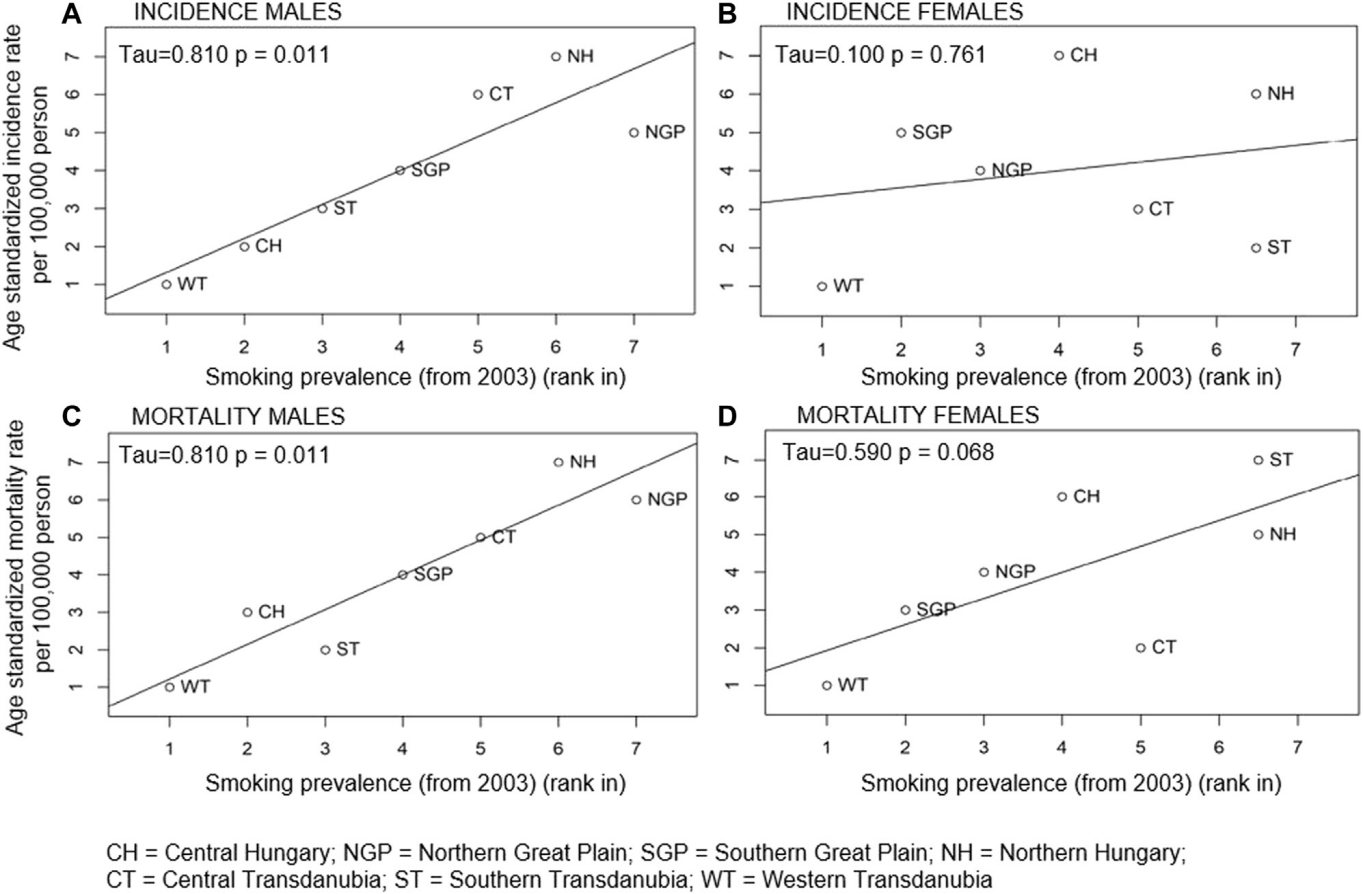
Correlation between within country rank in age-standardised LC incidence (A males, B females) and mortality rates (C males, D females) and smoking prevalence in the Hungarian regions in 2003.

We found a significant positive correlation between smoking prevalence and LC incidence and mortality rates among men. Higher smoking prevalence was associated with higher LC incidence and mortality rates (*p* = 0.01 for both incidence and mortality rates) as shown in Figure 4. However, the correlation between incidence rates of LC and smoking prevalence was not found to be significant in females (*p* = 0.8). As for mortality, the trend could be observed but was not statistically significant (*p* = 0.07).

### Relationship Between LC Incidence Rates and HDI, Levels of Income and Education and GDP per Capita in the Main Regions of Hungary

**FIGURE 5:**
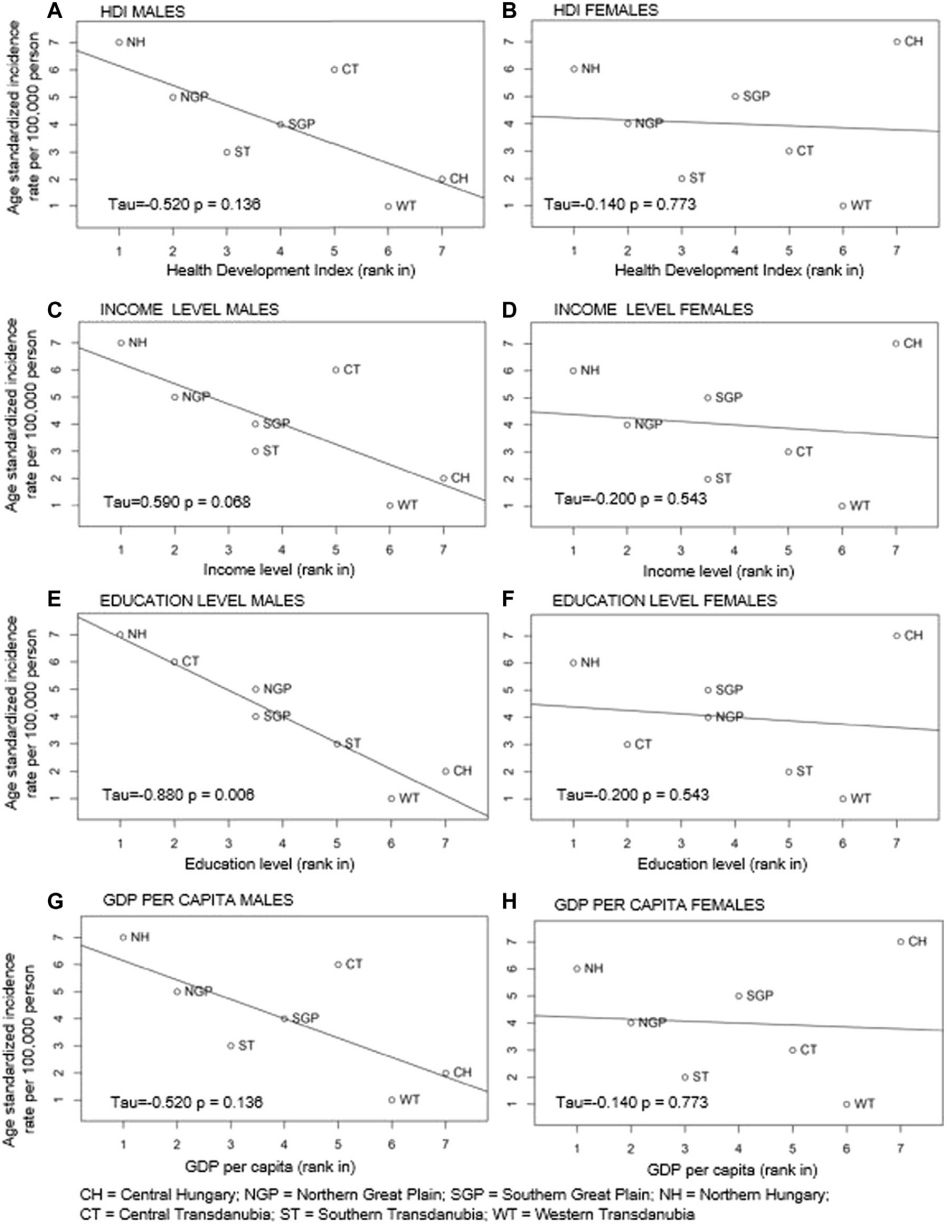
Correlations between ranked age-standardised LC incidence rates and Human Development Index (HDI) (A males; B females), level of income (C males; D females), level of education (E males; F females) and GDP per capita (G males; H females) in Hungary’s regions by gender.

LC incidence rates in men were inversely related to HDI, levels of education and income, and GDP per capita, although the association was statistically significant only in the case of education level (*p* = 0.006) (Figure 5).

In contrast, there were no indication of correlation between standardised LC incidence rates and HDI, income level, education level and GDP per capita in females in the Hungarian regions (*p* = 0.8, *p* = 0.5, *p* = 0.5, *p* = 0.8) (Figure 5).

Income and education levels were inversely correlated with smoking prevalence among males (*p* = 0.07 and *p* = 0.03), but not among females (*p* = 0.3 and *p* = 0.3) (Supplementary Table S3).

Supplementary Table S4 shows the ratio of LC-related mortality to total cancer mortality in Hungarian regions. Between 2011 and 2016, LC-related mortality accounted for 20–21% of total cancer mortality in men and for 14–15% of total cancer mortality in women. In both sexes, the ratios of LC-related mortality to total cancer mortality were the highest in the Northern and Eastern regions of the country.

## Discussion

LC mortality and incidence rates in Hungary were shown to be among the highest worldwide [1,2,3]. This emphasized the need for detailed and reliable epidemiological data on LC for the mapping of underlying regional characteristics. The present study analysed and compared epidemiological data and identified trends from the seven main regions of Hungary.

In line with international data, age-adjusted incidence and mortality rates in males were higher compared to females in all regions, which could be attributed to gender-differences in smoking habits [13]. The highest mortality and incidence rates were found in the Northern Hungarian and Northern Great Plain regions but showed a declining trend between 2011 and 2016. Central Hungary was the only exception, where age-adjusted mortality increased in males over the observation period, although this change was not significant. Our results are in line with earlier reports about the decreasing trend of male LC incidence in most Eastern European countries including Hungary [13,14]. We found strikingly high differences between regions in respect of incidence and mortality of LC. While rates among males in Northern Hungary and the Northern Great Plain were comparable to certain Eastern and South-Eastern European countries like Serbia and Macedonia, the lowest Hungarian incidence and mortality rates, recorded in Western Transdanubia, were similar to those found in certain Western European countries, such as the Netherlands and Denmark.

Previous studies have shown a strong correlation between lower smoking prevalence and a decline in male cancer rates in European countries [15,16]. Consequently, the apparent decreasing trends in lung cancer incidence and mortality among males in all Hungarian regions could be explained by the decline of smoking prevalence. Our correlation analysis demonstrated that the differences in lung cancer occurrence in 2013 were clearly related to smoking prevalence 10 years before. Furthermore, our analysis showed a correlation between lung cancer incidence and certain socioeconomic factors like education and income levels in males. Several publications have demonstrated the inverse correlations between lung cancer incidence and income, occupation, and educational level in both genders [17]. Furthermore, a recent Polish study reported an inverse association between socioeconomic status (SES) and LC mortality rates. It also showed that the population in Polish regions with lower SES had higher mortality rates due to LC and other respiratory diseases [18]. A study conducted in Spain also found a greater risk of LC-related death among men living in areas of greater socioeconomic deprivation [19]. Other studies found positive correlations between HDI, GDP per capita and lung cancer risk. A recent study showed that countries with higher World Health Organization (WHO) rankings and greater total expenditures on health/gross domestic product (e/GDP) had significantly higher crude LC incident and mortality rates and significantly higher age-standardised incident rates as well [20]. Wong et al. also reported higher LC incidence and mortality rates in countries with higher levels of HDI [8]. In line with the previously mentioned Polish and Spanish studies [18,19], the level of education in Hungary’s regions showed a significant inverse correlation with LC incidence among males, and the inverse relationship with the level of income was also detected. Although this trend of inverse correlation was likewise apparent between LC incidence and GDP per capita and HDI, these associations were not significant. Thus, high LC incidence in northern Hungary could be explained by the low education and income levels and the high smoking prevalence in these regions. Correlation analyses between education and income levels and smoking prevalence found significant inverse correlations in males, supporting our previous hypothesis. Additionally, lower education levels among people may lead to worse health literacy and decreased participation in screening programs, which can also play a role in higher lung cancer mortality.

A limitation of our study arises from its ecological design. As we used aggregated data by region, we could not study the independent effect of different factors including genetic factors [21] on the incidence of LC, as we had a limited number of observations (7 regions).

Gender differences are known to influence the incidence and outcomes of several diseases [22]. Increasing trends in cancer incidence and mortality rates including lung cancer among females in the Central European region have been reported by several previous publications [3,8,23]. Accordingly, an increasing trend in female age-adjusted incidence and mortality was apparent in all Hungarian regions, with the highest rates registered in Central Hungary.

As described earlier, a number of environmental and lifestyle factors have been associated with LC occurrence including tobacco smoking and industrialisation-related air pollution [24,25]. Studies have demonstrated a significant variability in LC incidence between metropolitan and non-metropolitan areas, with higher incidence rates found in metropolitan regions [12]. Based on our findings, the highest occurrence of LC in women in the Central Hungarian region may partly be attributed to the increasing prevalence of smoking among Hungarian women in urbanised cities. However, in contrast to males, no relevant correlations could be found between smoking prevalence and lung cancer incidence and mortality rates in females, neither could we detect correlations between lung cancer incidence rates and the examined socioeconomic factors. An important contributing factor, which is difficult to measure, may be the degree of urbanisation in Central Hungary. This region contains the largest metropolitan area of the nation, including the capital city, Budapest. Air pollution parameters were available from only some cities in Hungary, and the sporadic distribution of air quality monitoring stations could not provide a statistically relevant basis for the analysis of the connection between air pollution and LC incidence. Since the positive correlation between air pollution and lung cancer incidence has been proven by a number of studies, we conclude that air pollution may play an important role in the high LC incidence rates in the Central Hungarian region as well [26,27,28]. Air pollution probably plays a part in LC incidence in both genders, however, in males, the twice as high prevalence of smoking compared to females may overrule the impact of pollution. It would be important to conduct a study in the near future to examine these relationships at the level of settlements, where data about LC incidence and air pollution factors are both available.

The extremely high, up to 40% difference in rates of LC between the regions in both genders highlights the importance of mapping the underlying causes of these differences. Age-adjusted incidence and mortality rates were the lowest and declining in both genders during the study-period in Western Transdanubia, which is one of the most affluent regions in Hungary, highlighting the importance of socioeconomic factors in the smoking and lung cancer epidemic. Additional relevant characteristics of the region, e.g., lifestyle of the population, preventive programs, access to cessation services may help understanding the huge regional differences observed in our study. This is especially important considering the recent development of new and potent treatment options [29] and promising prognostic factors [30,31] for lung cancer which have the potential to significantly improve outcomes.

Our study found regional differences in the ratio of LC-related mortality to total cancer mortality in both sexes. In Northern and Eastern regions of Hungary, LC-related mortality accounted for higher percentages of total cancer deaths in both sexes compared to Central Hungary and Transdanubia. This may be explained by the generally poorer health status and higher cancer-related mortality of people living in Northern and Eastern Hungary as well as regional differences in health-related behaviours [32–34]. Specifically, Páldy et al. showed an accumulation of mortality from lung cancer in four Eastern counties of Hungary in both sexes [35]. Of note, in 2016, the highest death rate from cancer among people aged less than 65 years in the European Union was recorded in Northern Hungary [36]. Regional differences in smoking prevalence may also contribute to a larger proportion of LC-related deaths in regions lagging. Furthermore, North-Eastern Hungary has a high number of marginalised Roma communities who have poorer health status and higher smoking prevalence compared to the general population [37]. Although our study did not investigate potential differences in LC-related mortality between Roma communities and the general population, these factors may also have contributed to our results.

Our access to the NHIF database, which provides nationwide data, the large number of identified lung cancer patients and the 6-years follow-up period all provide a strong basis for our analysis. However, the NHIF database does not contain specific epidemiological data about lung cancer, such as pathomorphological data, tumour staging, laboratory findings and ECOG status as well as lung cancer screening rates.

## Conclusion

In conclusion, our analysis provides valuable information on regional differences in the epidemiology of LC within Hungary, a country with one of the highest reported LC incidence and mortality rates in the world.

Our study revealed considerable regional differences in LC incidence and mortality rates between the 7 main Hungarian regions. Although male LC incidence and mortality rates decreased over the study-period, the Northern Hungarian regions had particularly high incidence and mortality rates throughout the 6-years period. The increasing trend in LC incidence and mortality rates among females was most prominent in the highly urbanised Central Hungarian region. Understanding the causes of the huge regional differences may help develop more effective preventive measures.

## Data Availability

The original contributions presented in the study are included in the article/Supplementary Material, further inquiries can be directed to the corresponding author.

## Supplementary Material

Supplementary Figure S1 Age-standardised incidence and mortality rates (per 100.000 person years) among females in European countries and in the Hungarian regions based on the NHIF survey in 2012 (using European standard population from 1976).

Supplementary Figure S2 Incidence Rate Ratio by Regions in 2011 at males (A) and at females (B).

Supplementary Figure S3 Mortality Rate Ratio by Regions in 2011 at males (A) and females (B) and in 2016 at males (C) and females (D).

Supplementary Table S1 Age-standardised incidence rates by regions between 2011 and 2016.

Supplementary Table S2 Age-standardised mortality rates by regions between 2011 and 2016.

Supplementary Table S3 Correlation between age-standardised LC incidence and mortality rates and Human Development Index (HDI), income level, education level, GDP per Capita and smoking prevalence from 2003 in the main Hungarian regions. Correlation analysis between smoking prevalence (from 2003) and income and education levels.

Supplementary Table S4 Lung cancer mortality, total cancer mortality, and the ratio of lung cancer-related mortality to total cancer mortality in Hungarian regions between 2011 and 2016.
