# Supplementary figures and images for: Significant Regional Differences in Lung Cancer Incidence in Hungary: Epidemiological Study Between 2011 and 2016
Source: Pathol Oncol Res. 2021 Sep 14;27:1609916. doi: 10.3389/pore.2021.1609916 (PMC8478017; doi:10.3389/pore.2021.1609916)

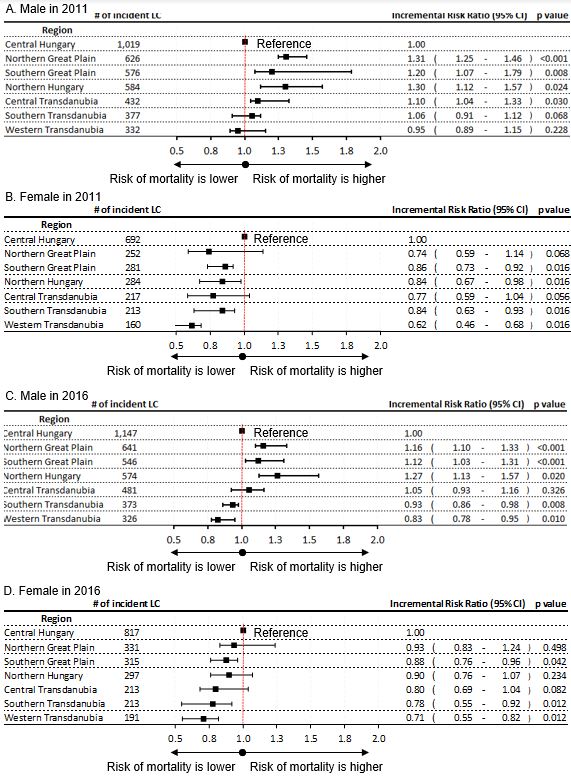

Supplement: Supplementary file 1 [file image3.jpg]

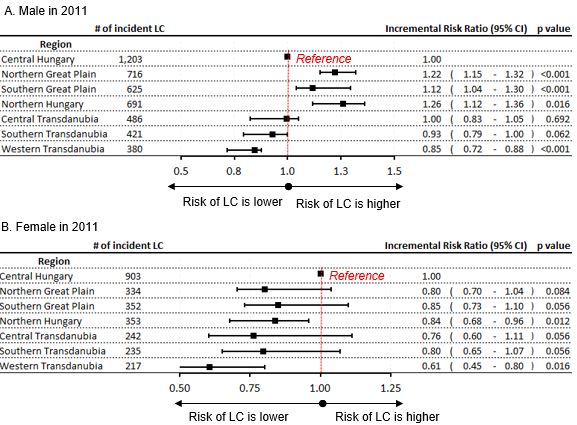

Supplement: Supplementary file 2 [file image2.jpg]

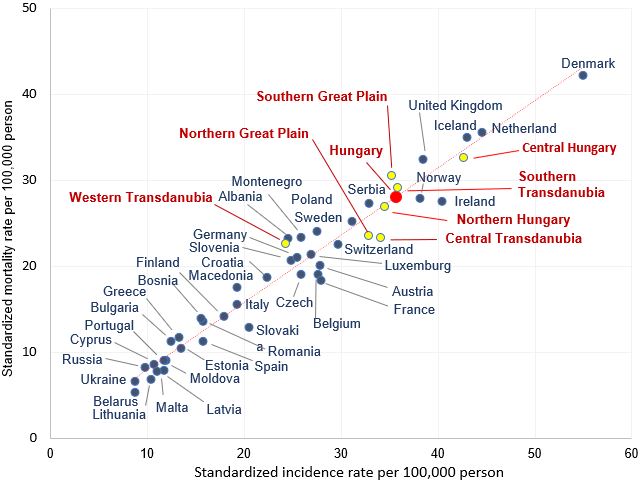

Supplement: Supplementary file 7 [file image1.jpg]
