# Supplementary material for: Significant Regional Differences in Lung Cancer Incidence in Hungary: Epidemiological Study Between 2011 and 2016
Source: Pathol Oncol Res. 2021 Sep 14;27:1609916. doi: 10.3389/pore.2021.1609916 (PMC8478017; doi:10.3389/pore.2021.1609916)
Supplement: Supplementary file 3 [file table1.docx]

| **Incidence Rate per 100,000 Person Years** | | | | | | | | | | | | | |  |  |  |  |  |  |  |  |
| --- | --- | --- | --- | --- | --- | --- | --- | --- | --- | --- | --- | --- | --- | --- | --- | --- | --- | --- | --- | --- | --- |
| **Characteristics** | 2011 | 2012 | 2013 | 2014 | 2015 | 2016 | Mean Annual Change | | | | | | |  | Change between 2011 and 2016 | | | | | | |
|  |  |  |  |  |  |  | % |  | 95% CI | | |  | p value |  | % |  | 95% CI |  | 95% CI |  | p value |
| **Overall** | **75.34** | **71.74** | **70.98** | **71.23** | **71.05** | **70.67** | **-0.90%** | ( | **-2.49%** | **-** | **0.00%** | **)** | **0.090** |  | **-4.42%** | ( | **-11.85%** | **-** | **0.00%** | **)** | **0.090** |
| Central Hungary | 77.05 | 76.93 | 74.03 | 72.31 | 71.33 | 73.50 | -1.39% | ( | -3.42% | - | 0.90% | ) | 0.084 |  | -6.76% | ( | -15.95% | - | 4.60% | ) | 0.084 |
| Northern Great Plain | 78.04 | 78.40 | 77.37 | 72.96 | 73.48 | 69.91 | -2.26% | ( | -3.12% | - | -0.75% | ) | <0.001 |  | -10.82% | ( | -14.67% | - | -3.71% | ) | <0.001 |
| Southern Great Plain | 75.81 | 73.34 | 75.73 | 76.91 | 75.18 | 69.86 | -0.80% | ( | -4.25% | - | 2.93% | ) | 0.504 |  | -3.94% | ( | -19.50% | - | 15.54% | ) | 0.504 |
| Northern Hungary | 90.52 | 75.52 | 77.74 | 82.00 | 73.11 | 82.67 | -1.41% | ( | -6.09% | - | 3.68% | ) | 0.374 |  | -6.85% | ( | -26.97% | - | 19.78% | ) | 0.374 |
| Central Transdanubia | 71.26 | 69.91 | 68.08 | 70.30 | 73.95 | 72.03 | 0.81% | ( | -2.00% | - | 4.10% | ) | 0.226 |  | 4.12% | ( | -9.61% | - | 22.27% | ) | 0.226 |
| Southern Transdanubia | 57.47 | 65.97 | 63.45 | 64.87 | 71.44 | 64.42 | 2.29% | ( | -1.45% | - | 6.68% | ) | 0.106 |  | 11.97% | ( | -7.06% | - | 38.17% | ) | 0.106 |
| Western Transdanubia | 61.90 | 55.88 | 54.18 | 57.26 | 59.88 | 60.36 | 0.38% | ( | -3.64% | - | 5.38% | ) | 0.990 |  | 1.92% | ( | -16.92% | - | 29.95% | ) | 0.990 |
| **Male LC patients** | **115.10** | **107.49** | **102.72** | **104.31** | **100.94** | **100.94** | -2.27% | ( | **-3.77%** | **-** | **-0.81%** | **)** | **0.008** |  | **-10.84%** | ( | **-17.49%** | **-** | **-3.97%** | **)** | **0.008** |
| Central Hungary | 108.11 | 103.62 | 98.47 | 97.85 | 95.84 | 93.00 | -2.77% | ( | -4.53% | - | -1.20% | ) | 0.008 |  | -13.12% | ( | -20.71% | - | -5.86% | ) | 0.008 |
| Northern Great Plain | 128.99 | 131.16 | 114.00 | 111.86 | 109.58 | 106.24 | -4.35% | ( | -8.74% | - | -1.77% | ) | 0.008 |  | -19.95% | ( | -36.68% | - | -8.55% | ) | 0.008 |
| Southern Great Plain | 117.17 | 114.14 | 113.88 | 119.08 | 107.56 | 104.95 | -1.78% | ( | -5.60% | - | 1.81% | ) | 0.182 |  | -8.60% | ( | -25.02% | - | 9.36% | ) | 0.182 |
| Northern Hungary | 146.79 | 122.79 | 121.62 | 128.60 | 110.21 | 126.94 | -2.77% | ( | -7.89% | - | 1.87% | ) | 0.162 |  | -13.12% | ( | -33.69% | - | 9.72% | ) | 0.162 |
| Central Transdanubia | 115.33 | 103.84 | 103.33 | 101.98 | 108.30 | 113.37 | 0.09% | ( | -4.10% | - | 5.13% | ) | 0.966 |  | 0.47% | ( | -18.87% | - | 28.41% | ) | 0.966 |
| Southern Transdanubia | 108.47 | 97.10 | 90.34 | 92.59 | 102.07 | 95.66 | -1.07% | ( | -7.25% | - | 6.00% | ) | 0.528 |  | -5.24% | ( | -31.36% | - | 33.81% | ) | 0.528 |
| Western Transdanubia | 94.72 | 89.99 | 84.22 | 84.36 | 84.19 | 90.19 | -1.38% | ( | -5.82% | - | 2.94% | ) | 0.300 |  | -6.71% | ( | -25.91% | - | 15.60% | ) | 0.300 |
| **Female LC patients** | **48.16** | **47.20** | **49.31** | **48.78** | **50.76** | **50.09** | 1.34% | ( | **0.49%** | **-** | **2.52%** | **)** | **0.024** |  | **6.86%** | ( | **2.50%** | **-** | **13.25%** | **)** | **0.024** |
| Central Hungary | 56.04 | 58.31 | 58.10 | 54.34 | 54.10 | 59.86 | 0.06% | ( | -4.05% | - | 4.58% | ) | 0.716 |  | 0.30% | ( | -18.70% | - | 25.09% | ) | 0.716 |
| Northern Great Plain | 43.48 | 43.36 | 52.41 | 46.78 | 48.93 | 45.84 | 1.76% | ( | -3.52% | - | 8.63% | ) | 0.298 |  | 9.11% | ( | -16.39% | - | 51.27% | ) | 0.298 |
| Southern Great Plain | 47.64 | 46.16 | 49.61 | 48.67 | 53.66 | 46.92 | 1.17% | ( | -1.40% | - | 5.12% | ) | 0.464 |  | 5.97% | ( | -6.78% | - | 28.38% | ) | 0.464 |
| Northern Hungary | 53.14 | 44.30 | 47.56 | 51.24 | 49.04 | 55.24 | 1.81% | ( | -2.84% | - | 7.86% | ) | 0.190 |  | 9.37% | ( | -13.42% | - | 45.98% | ) | 0.190 |
| Central Transdanubia | 41.48 | 46.11 | 43.47 | 48.16 | 51.67 | 44.80 | 2.76% | ( | -1.06% | - | 9.60% | ) | 0.066 |  | 14.59% | ( | -5.21% | - | 58.15% | ) | 0.066 |
| Southern Transdanubia | 44.18 | 45.48 | 45.14 | 45.92 | 49.74 | 43.32 | 0.75% | ( | -1.20% | - | 4.92% | ) | 0.538 |  | 3.82% | ( | -5.88% | - | 27.17% | ) | 0.538 |
| Western Transdanubia | 39.41 | 32.15 | 33.56 | 38.62 | 43.49 | 39.59 | 3.66% | ( | -4.90% | - | 11.94% | ) | 0.102 |  | 19.7% | ( | -22.2% | - | 75.8% | ) | 0.102 |
