# Supplementary material for: Significant Regional Differences in Lung Cancer Incidence in Hungary: Epidemiological Study Between 2011 and 2016
Source: Pathol Oncol Res. 2021 Sep 14;27:1609916. doi: 10.3389/pore.2021.1609916 (PMC8478017; doi:10.3389/pore.2021.1609916)
Supplement: Supplementary file 4 [file table2.docx]

| **Mortality Rate per 100,000 Person years** | | | | | | | | | |  |  |  |  |  |  |  |  |  |  |  |  |
| --- | --- | --- | --- | --- | --- | --- | --- | --- | --- | --- | --- | --- | --- | --- | --- | --- | --- | --- | --- | --- | --- |
| **Characteristics** | 2011 | 2012 | 2013 | 2014 | 2015 | 2016 | Mean Annual Change | | | | | | |  | Change between 2011 and 2016 | | | | | | |
|  |  |  |  |  |  |  | % |  | 95% CI | | |  | p value |  | Change, % |  | 95% CI |  | 95% CI |  | p value |
| **Overall** | **64.4** | **65.1** | **64.4** | **65.0** | **64.5** | **66.0** | 0.47% | ( | **-0.27%** | **-** | **0.94%** | **)** | **0.082** |  | **2.39%** | **(** | **-1.4%** | **-** | **4.8%** | **)** | **0.082** |
| Central Hungary | 63.4 | 67.8 | 66.0 | 65.1 | 65.7 | 68.7 | 0.89% | ( | -2.36% | - | 3.25% | ) | 0.214 |  | 4.54% | ( | -11.3% | - | 17.4% | ) | 0.214 |
| Northern Great Plain | 66.7 | 68.7 | 70.5 | 72.0 | 68.7 | 70.1 | 0.70% | ( | -1.32% | - | 2.34% | ) | 0.212 |  | 3.54% | ( | -6.4% | - | 12.3% | ) | 0.212 |
| Southern Great Plain | 67.5 | 69.5 | 66.8 | 68.7 | 70.3 | 65.2 | -0.39% | ( | -2.12% | - | 2.07% | ) | 0.450 |  | -1.94% | ( | -10.2% | - | 10.8% | ) | 0.450 |
| Northern Hungary | 76.1 | 68.5 | 69.5 | 76.6 | 69.6 | 75.4 | 0.34% | ( | -3.19% | - | 5.00% | ) | 0.838 |  | 1.70% | ( | -15.0% | - | 27.7% | ) | 0.838 |
| Central Transdanubia | 63.7 | 63.2 | 62.5 | 64.7 | 60.2 | 65.6 | 0.18% | ( | -1.59% | - | 2.17% | ) | 0.562 |  | 0.91% | ( | -7.7% | - | 11.3% | ) | 0.562 |
| Southern Transdanubia | 52.2 | 63.5 | 60.2 | 56.1 | 60.8 | 61.4 | 1.33% | ( | -6.41% | - | 5.06% | ) | 0.428 |  | 6.85% | ( | -28.2% | - | 28.0% | ) | 0.428 |
| Western Transdanubia | 52.1 | 52.9 | 53.0 | 50.4 | 53.6 | 51.6 | -0.04% | ( | -2.33% | - | 2.18% | ) | 0.900 |  | -0.21% | ( | -11.1% | - | 11.4% | ) | 0.900 |
| **Male LC patients** | **103.8** | **104.8** | **101.5** | **101.2** | **97.2** | **102.6** | -0.86% | ( | **-2.39%** | **-** | **0.49%** | **)** | **0.126** |  | **-4.22%** | **(** | **-11.4%** | **-** | **2.5%** | **)** | **0.126** |
| Central Hungary | 94.5 | 98.0 | 94.0 | 91.4 | 94.8 | 99.5 | 0.30% | ( | -3.72% | - | 4.76% | ) | 0.524 |  | 1.50% | ( | -17.3% | - | 26.2% | ) | 0.524 |
| Northern Great Plain | 119.0 | 124.1 | 120.6 | 113.9 | 108.0 | 114.0 | -2.06% | ( | -5.49% | - | 0.96% | ) | 0.080 |  | -9.89% | ( | -24.6% | - | 4.9% | ) | 0.080 |
| Southern Great Plain | 112.4 | 112.9 | 105.2 | 116.7 | 108.9 | 103.1 | -1.10% | ( | -6.08% | - | 3.42% | ) | 0.294 |  | -5.37% | ( | -26.9% | - | 18.3% | ) | 0.294 |
| Northern Hungary | 129.2 | 119.3 | 117.5 | 127.5 | 111.1 | 128.5 | -0.26% | ( | -4.74% | - | 3.05% | ) | 0.786 |  | -1.30% | ( | -21.6% | - | 16.2% | ) | 0.786 |
| Central Transdanubia | 101.3 | 107.7 | 104.5 | 101.3 | 88.6 | 110.9 | -0.53% | ( | -7.05% | - | 2.77% | ) | 0.840 |  | -2.60% | ( | -30.6% | - | 14.7% | ) | 0.840 |
| Southern Transdanubia | 99.6 | 102.7 | 95.9 | 85.4 | 91.1 | 95.8 | -2.14% | ( | -8.63% | - | 6.11% | ) | 0.152 |  | -10.27% | ( | -36.3% | - | 34.5% | ) | 0.152 |
| Western Transdanubia | 86.8 | 87.7 | 87.2 | 83.4 | 79.7 | 78.0 | -2.51% | ( | -4.03% | - | -1.11% | ) | 0.032 |  | -11.94% | ( | -18.6% | - | -5.4% | ) | 0.032 |
| **Female LC patients** | **38.3** | **38.7** | **39.6** | **40.8** | **42.7** | **41.6** | 2.38% | ( | **1.71%** | **-** | **3.49%** | **)** | **0.016** |  | **12.49%** | **(** | **8.9%** | **-** | **18.7%** | **)** | **0.016** |
| Central Hungary | 42.9 | 47.2 | 47.8 | 46.6 | 45.5 | 48.0 | 1.20% | ( | -2.10% | - | 4.36% | ) | 0.446 |  | 6.15% | ( | -10.1% | - | 23.8% | ) | 0.446 |
| Northern Great Plain | 32.8 | 32.7 | 37.1 | 44.5 | 42.2 | 41.1 | 6.02% | ( | -4.22% | - | 16.31% | ) | 0.068 |  | 33.98% | ( | -19.4% | - | 112.9% | ) | 0.068 |
| Southern Great Plain | 37.8 | 41.2 | 41.4 | 37.7 | 44.8 | 40.9 | 1.63% | ( | -3.01% | - | 6.06% | ) | 0.174 |  | 8.41% | ( | -14.2% | - | 34.2% | ) | 0.174 |
| Northern Hungary | 42.5 | 36.1 | 38.0 | 43.4 | 43.1 | 43.3 | 2.46% | ( | -0.09% | - | 8.79% | ) | 0.076 |  | 12.94% | ( | -0.4% | - | 52.4% | ) | 0.076 |
| Central Transdanubia | 37.2 | 33.3 | 33.9 | 39.6 | 41.3 | 35.0 | 1.89% | ( | -5.50% | - | 10.80% | ) | 0.486 |  | 9.79% | ( | -24.6% | - | 67.0% | ) | 0.486 |
| Southern Transdanubia | 40.2 | 38.9 | 36.2 | 36.0 | 40.6 | 38.3 | -0.26% | ( | -4.68% | - | 5.72% | ) | 0.568 |  | -1.28% | ( | -21.3% | - | 32.1% | ) | 0.568 |
| Western Transdanubia | 29.1 | 30.2 | 29.5 | 28.3 | 36.6 | 33.7 | 4.04% | ( | -2.74% | - | 12.77% | ) | 0.208 |  | 21.90% | ( | -13.0% | - | 82.4% | ) | 0.208 |
