# Supplementary material for: Significant Regional Differences in Lung Cancer Incidence in Hungary: Epidemiological Study Between 2011 and 2016
Source: Pathol Oncol Res. 2021 Sep 14;27:1609916. doi: 10.3389/pore.2021.1609916 (PMC8478017; doi:10.3389/pore.2021.1609916)
Supplement: Supplementary file 5 [file table3.docx]

| Standardized rate per 100.000 population | Gender | HDI | | Income | | Education | | GDP per capita | | Smoking prevalence | |
| --- | --- | --- | --- | --- | --- | --- | --- | --- | --- | --- | --- |
|  |  | Tau | p value | Tau | p value | Tau | p value | Tau | p value | Tau | p value |
| Incidence rate | Female | -0.140 | 0.773 | -0.200 | 0.543 | -0.200 | 0.543 | -0.140 | 0.773 | 0.100 | 0.761 |
|  | Male | -0.520 | 0.136 | -0.590 | 0.068 | -0.880 | 0.006 | -0.520 | 0.136 | 0.810 | 0.011 |
| Mortality rate | Female | -0.330 | 0.381 | -0.290 | 0.362 | 0.100 | 0.761 | -0.330 | 0.381 | 0.590 | 0.068 |
|  | Male | -0.520 | 0.136 | -0.590 | 0.068 | -0.680 | 0.033 | -0.520 | 0.136 | 0.810 | 0.011 |
|  | Gender | Income | | Education | |  |  |  |  |  |  |
|  |  | Tau | p value | Tau | p value |  |  |  |  |  |  |
| Smoking prevalence | Female | -0.350 | 0.282 | -0.350 | 0.282 |  |  |  |  |  |  |
|  | Male | -0.590 | 0.068 | -0.680 | 0.033 |  |  |  |  |  |  |
