# Supplementary material for: Significant Regional Differences in Lung Cancer Incidence in Hungary: Epidemiological Study Between 2011 and 2016
Source: Pathol Oncol Res. 2021 Sep 14;27:1609916. doi: 10.3389/pore.2021.1609916 (PMC8478017; doi:10.3389/pore.2021.1609916)
Supplement: Supplementary file 6 [file table4.docx]

|  | **Lung Cancer Mortality Rate per 100,000 Person years** | | | | | |  | **All Cancer Mortality Rate per 100,000 Person years** | | | | | |  | **Lung Cancer versus All cancer mortality** | | | | | |
| --- | --- | --- | --- | --- | --- | --- | --- | --- | --- | --- | --- | --- | --- | --- | --- | --- | --- | --- | --- | --- |
| **Characteristics** | 2011 | 2012 | 2013 | 2014 | 2015 | 2016 |  | 2011 | 2012 | 2013 | 2014 | 2015 | 2016 |  | 2011 | 2012 | 2013 | 2014 | 2015 | 2016 |
|  |  |  |  |  |  |  |  |  |  |  |  |  |  |  |  |  |  |  |  |  |
| **Overall** | **64.4** | **65.1** | **64.4** | **65.0** | **64.5** | **66.0** |  | **364.9** | **367.8** | **358.9** | **354.7** | **351.9** | **352.3** |  | **17.66%** | **17.70%** | **17.95%** | **18.32%** | **18.33%** | **18.73%** |
| Central Hungary | 63.4 | 67.8 | 66.0 | 65.1 | 65.7 | 68.7 |  | 360.7 | 362.8 | 352.3 | 336.8 | 340.6 | 340.1 |  | 17.57% | 18.68% | 18.74% | 19.32% | 19.30% | 20.22% |
| Northern Great Plain | 66.7 | 68.7 | 70.5 | 72.0 | 68.7 | 70.1 |  | 365.9 | 376.6 | 352.3 | 363.8 | 359.6 | 357.8 |  | 18.23% | 18.25% | 20.01% | 19.80% | 19.12% | 19.58% |
| Southern Great Plain | 67.5 | 69.5 | 66.8 | 68.7 | 70.3 | 65.2 |  | 349.7 | 363.9 | 354.5 | 347.5 | 347.8 | 348.7 |  | 19.30% | 19.09% | 18.83% | 19.77% | 20.21% | 18.71% |
| Northern Hungary | 76.1 | 68.5 | 69.5 | 76.6 | 69.6 | 75.4 |  | 330.2 | 326.5 | 319.2 | 324.0 | 314.1 | 311.7 |  | 23.06% | 20.98% | 21.78% | 23.66% | 22.17% | 24.19% |
| Central Transdanubia | 63.7 | 63.2 | 62.5 | 64.7 | 60.2 | 65.6 |  | 376.6 | 372.6 | 370.7 | 362.1 | 365.0 | 371.6 |  | 16.92% | 16.98% | 16.85% | 17.87% | 16.48% | 17.66% |
| Southern Transdanubia | 52.2 | 63.5 | 60.2 | 56.1 | 60.8 | 61.4 |  | 374.3 | 364.2 | 375.5 | 374.4 | 374.5 | 363.8 |  | 13.94% | 17.44% | 16.04% | 14.98% | 16.24% | 16.89% |
| Western Transdanubia | 52.1 | 52.9 | 53.0 | 50.4 | 53.6 | 51.6 |  | 353.0 | 361.8 | 352.4 | 350.6 | 328.8 | 340.2 |  | 14.75% | 14.63% | 15.03% | 14.38% | 16.31% | 15.17% |
| **Male LC patients** | **103.8** | **104.8** | **101.5** | **101.2** | **97.2** | **102.6** |  | **510.6** | **514.3** | **497.2** | **486.7** | **479.6** | **488.6** |  | **20.32%** | **20.37%** | **20.41%** | **20.80%** | **20.26%** | **20.99%** |
| Central Hungary | 94.5 | 98.0 | 94.0 | 91.4 | 94.8 | 99.5 |  | 480.1 | 470.0 | 460.3 | 438.6 | 433.3 | 450.7 |  | 19.69% | 20.85% | 20.43% | 20.85% | 21.88% | 22.08% |
| Northern Great Plain | 119.0 | 124.1 | 120.6 | 113.9 | 108.0 | 114.0 |  | 533.6 | 558.0 | 513.3 | 519.3 | 523.7 | 510.0 |  | 22.30% | 22.24% | 23.49% | 21.93% | 20.63% | 22.35% |
| Southern Great Plain | 112.4 | 112.9 | 105.2 | 116.7 | 108.9 | 103.1 |  | 486.5 | 515.5 | 489.2 | 480.2 | 481.9 | 471.6 |  | 23.10% | 21.90% | 21.50% | 24.31% | 22.61% | 21.87% |
| Northern Hungary | 129.2 | 119.3 | 117.5 | 127.5 | 111.1 | 128.5 |  | 554.3 | 540.2 | 524.5 | 520.2 | 508.8 | 503.2 |  | 23.31% | 22.09% | 22.41% | 24.51% | 21.83% | 25.54% |
| Central Transdanubia | 101.3 | 107.7 | 104.5 | 101.3 | 88.6 | 110.9 |  | 545.3 | 535.2 | 526.4 | 511.7 | 492.0 | 528.9 |  | 18.58% | 20.12% | 19.86% | 19.80% | 18.01% | 20.97% |
| Southern Transdanubia | 99.6 | 102.7 | 95.9 | 85.4 | 91.1 | 95.8 |  | 505.6 | 520.4 | 516.4 | 509.7 | 518.0 | 516.3 |  | 19.71% | 19.73% | 18.56% | 16.75% | 17.59% | 18.56% |
| Western Transdanubia | 86.8 | 87.7 | 87.2 | 83.4 | 79.7 | 78.0 |  | 497.0 | 503.1 | 493.4 | 487.3 | 452.9 | 487.5 |  | 17.47% | 17.43% | 17.68% | 17.13% | 17.59% | 16.00% |
| **Female LC patients** | **38.3** | **38.7** | **39.6** | **40.8** | **42.7** | **41.6** |  | **273.6** | **276.5** | **273.4** | **272.2** | **272.1** | **267.5** |  | **13.98%** | **13.99%** | **14.49%** | **15.00%** | **15.69%** | **15.54%** |
| Central Hungary | 42.9 | 47.2 | 47.8 | 46.6 | 45.5 | 48.0 |  | 288.3 | 297.6 | 287.2 | 273.9 | 283.6 | 272.6 |  | 14.87% | 15.85% | 16.65% | 17.01% | 16.05% | 17.60% |
| Northern Great Plain | 32.8 | 32.7 | 37.1 | 44.5 | 42.2 | 41.1 |  | 259.9 | 260.6 | 251.2 | 266.4 | 256.1 | 262.0 |  | 12.64% | 12.54% | 14.79% | 16.72% | 16.48% | 15.67% |
| Southern Great Plain | 37.8 | 41.2 | 41.4 | 37.7 | 44.8 | 40.9 |  | 263.4 | 268.8 | 270.2 | 264.9 | 265.4 | 271.5 |  | 14.36% | 15.34% | 15.31% | 14.23% | 16.87% | 15.08% |
| Northern Hungary | 42.5 | 36.1 | 38.0 | 43.4 | 43.1 | 43.3 |  | 263.6 | 265.3 | 263.3 | 275.8 | 263.4 | 262.3 |  | 16.12% | 13.60% | 14.43% | 15.75% | 16.38% | 16.53% |
| Central Transdanubia | 37.2 | 33.3 | 33.9 | 39.6 | 41.3 | 35.0 |  | 268.2 | 271.2 | 271.3 | 267.3 | 282.9 | 273.3 |  | 13.88% | 12.28% | 12.49% | 14.80% | 14.60% | 12.81% |
| Southern Transdanubia | 40.2 | 38.9 | 36.2 | 36.0 | 40.6 | 38.3 |  | 287.6 | 264.5 | 287.2 | 286.0 | 284.3 | 265.4 |  | 13.99% | 14.70% | 12.60% | 12.60% | 14.28% | 14.41% |
| Western Transdanubia | 29.1 | 30.2 | 29.5 | 28.3 | 36.6 | 33.7 |  | 257.1 | 268.5 | 261.3 | 264.5 | 248.1 | 249.5 |  | 11.30% | 11.26% | 11.30% | 10.71% | 14.73% | 13.52% |
